# Effect of ultrasound on the physicochemical, mechanical and adhesive properties of micro-arc oxidized coatings on Ti13Nb13Zr bio-alloy

**DOI:** 10.1038/s41598-024-75626-4

**Published:** 2024-10-25

**Authors:** Balbina Makurat-Kasprolewicz, Marcin Wekwejt, Luca Pezzato, Anna Ronowska, Jolanta Krupa, Sławomir Zimowski, Stefan Dzionk, Agnieszka Ossowska

**Affiliations:** 1 Department of Materials Science and Technology, Gdańsk University of Technology, Gdańsk, 80-233 Poland; 2 Department of Machine Design and Medical Engineering, Gdańsk University of Technology, Gdańsk, 80-233 Poland; 3 Department of Biomaterials Technology, Gdańsk University of Technology, Gdańsk, 80-233 Poland; 4 Institute of Condensed Matter Chemistry and Energy Technologies, National Research Council of Italy, Padova, 35127 Italy; 5 Department of Laboratory Medicine, Medical University of Gdańsk, Gdańsk, 80-210 Poland; 6 Department of Machine Design and Maintenance, AGH University of Kraków, Kraków, 30-059 Poland; 7 Department of Manufacturing and Production Engineering, Gdańsk University of Technology, Gdańsk, 80-233 Poland

**Keywords:** Plasma electrolytic oxidation, Ultrasound, Titanium alloy, Nanoindentation, Adhesion, Biomedical application, Engineering, Materials science

## Abstract

Implant surgeries are increasingly challenging due to their rising number. Achieving the desired biomaterial surface properties to ensure a strong bond with human tissue is a significant issue. This study investigates the influence of ultrasound (US) during the micro-arc oxidation (MAO) process on Ti13Zr13Nb bio-alloy, an area not previously explored, to enhance titanium alloy coatings’ properties for biomedical applications. Porous calcium-phosphate-based coatings were successfully deposited on Ti13Zr13Nb using MAO and ultrasound micro-arc oxidation (UMAO). Various properties such as morphology, chemical composition, topography, wettability, surface free energy, thickness, adhesion to the substrate, as well as mechanical and corrosion characteristics were thoroughly analyzed. Cytocompatibility was assessed using human osteoblasts. Using US during the MAO process increased coating roughness (up to ~ 17%), core height (up to 22%), isotropy (up to 17%), thickness (up to ~ 46%), and hardness (up to ~ 18%), depending on MAO parameters and US mode. Optimal coating performance was achieved at 136 mA, 600 s, and a sinusoidal US setting, resulting in the highest isotropy (~ 79%) and rutile quantity (2.6%), the lowest elastic modulus (~ 57 GPa), and the contact angle of ~ 70°, all of which could have contributed to enhancing osteoblast viability in vitro. This study, for the first time, underscores the importance of using the US during the MAO in tailoring the Ti13Zr13Nb for specific biomedical applications.

**Supplementary Information:**

The online version contains supplementary material available at 10.1038/s41598-024-75626-4.

## Introduction

The increasing proportion of elderly people in society, the growing number of road accidents, and a sedentary lifestyle lead to an expanding number of bone injuries, contributing to continuous attempts to develop hard tissue engineering and materials used in implantology^1^. Particularly noteworthy is the increasingly popular near β type titanium alloy Ti13Zr13Nb, used in this study, due to its superior biocompatibility, low elastic modulus similar to that of bone, and high tensile and yield strength, making it ideal for biomedical applications compared to other Ti-based alloys^2–4^. These features are crucial when selecting materials for hard tissue engineering, such as prosthetic joints or artificial bones^5^. However, despite its many advantages, this material still has challenges: it is not bioactive without adequate surface preparation and has relatively poor corrosion resistance in human body fluids, which significantly influences implant rejection^1,6^.

Current research primarily focuses on modifying implant surfaces to amend and strengthen the formation of the tissue-implant interface. Additionally, the generated coatings are being optimized to exhibit suitable physicochemical and wear properties^7^. One of the processes enabling the modification of the surface of implants is plasma electrolytic oxidation (PEO), also called micro-arc oxidation (MAO). This electrochemical process generates porous coatings that mimic the structure of bone^8^. Depending on the applied voltage, current, process time, and electrolyte composition, it is possible to adjust the characteristics of the coatings (such as morphology, chemical composition, crystallinity, thickness, roughness, mechanical and tribological properties, adhesion to the substrate, corrosion resistance, surface wettability, surface free energy, cytocompatibility, antibacterial properties, etc.)^6,9,10^. MAO coatings generally have beneficial hydrophilicity and corrosion resistance for biomedical applications, and their chemical composition can be tailored by changing electrolyte components^6,11^. At the same time, these coatings can be characterized by non-uniform morphology because, during the process, a mullite phase accumulates around the discharge channels^12^. This may contribute to obtaining poor-quality coatings and, consequently, to premature failure of the implant^13^. The utilization of ultrasound (US) in different surface treatment methods is attributed to its capability to enhance the mass transfer process and ensure a more even distribution of coatings^14^. When combined with MAO, US can contribute to the formation of a ceramic coating on the titanium surface that is both more consistent and cohesive^15^. This enhanced coating not only improves the material’s wear resistance but also ameliorates the adherence between the ceramic coating and the substrate^16^.

In our previous work^13^, US was successfully applied in the MAO process on commercially pure titanium, and the results indicated that depending on US mode, it could increase coating thickness, isotropy, roughness, skewness, and calcium incorporation, as well as improve porosity and pore size, which consequently may redound to the greatest adhesion and proliferation of osteoblasts. Xu et al.^17^ demonstrated that the implementation of US during the MAO process on Ti6Al4V alloy impacts the morphology, phase composition, wear resistance, and corrosion properties of coatings. Another study by Kazantsevaet al.^18^ on commercially pure titanium confirmed that the power of US influences morphology as well as elemental and phase composition of the micro-arc oxidized coatings. The study conducted by Shen et al.^19^ showcased that the utilization of US during the MAO process on 6061Al alloy resulted in a reduction of the dielectric breakdown voltage of the coating while simultaneously enhancing the rate of coating growth.

**Fig. 1:**
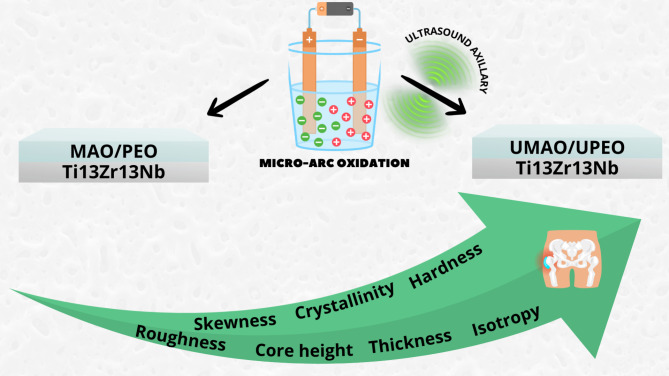
Schematic presentation of ultrasound effects on micro-arc oxidized coating properties on Ti13Zr13Nb alloy.

Regarding the above reports, the authors of the study, for the first time, examined the impact of the US in the MAO process (schematically presented in Fig. 1) on fundamental structural properties such as morphology, chemical and phase composition, and coating thickness. A comprehensive analysis is presented, specifically covering the topography, contact angle, and surface energy of the coatings. Additionally, the study aimed to evaluate the effects of the US on mechanical factors (hardness and elastic modulus) as well as the adhesion to the substrate. This detailed examination provides valuable insights into properties that are seldom addressed in existing literature yet play a crucial role in determining the implant’s lifespan. The investigation was conducted on a titanium alloy, specifically Ti13Zr13Nb, which was chosen because it possesses favorable attributes for usage in biomedical applications, including beneficial biocompatibility and advantageous mechanical properties^20^. The surface was modified using the MAO and ultrasound micro-arc oxidation (UMAO) techniques and these processes involved an electrolyte containing calcium acetate hydrate and β-glycerophosphate disodium salt pentahydrate, both serving as sources of calcium and phosphorus. This choice was based on the known stimulation of robust connections between implants and bones through the presence of these elements since calcium and phosphorus participate in the osteointegration of bone with the implant and constitute the basic building blocks of bone, which facilitates the enhancement of the modified material’s biocompatibility^20,21^. Moreover, their addition can enhance the wear resistance properties of micro-arc oxidized alloys^22^.

## Experimental section

### Specimens Preparation

For the study, Ti13Nb13Zr titanium bio-alloy (Table 1) discs were utilized. These specimens were circular disks, measuring approximately 4 mm in thickness and 14 mm in diameter, obtained by cutting from homogeneous bars. Prior to the modifications, the substrates underwent a polishing process using SiC abrasive papers with grit sizes of #120, #500, #800, and #1200. Subsequently, the specimens were subjected to ultrasonic cleaning and degreasing using methanol (p.a.; STANLAB, Poland), isopropanol (p.a.; EUROCHEM BGD, Poland), and distilled water, each for a duration of 5 min. Following the ultrasonic treatment, the specimens were dried using compressed air and then left in ambient air.

**Table 1:** Chemical composition of Ti13Zr13Nb alloy by weight% (based on mill test certificate no: 20221130).

Element	Ti	Nb	Zr	Fe	C	*N*	O	H
Weight%	Balance	12.98	13.20	0.003	0.006	0.02	0.06	0.005

### Micro-arc oxidation process

The MAO and UMAO procedures were conducted using a DC power supply (MR 1000020, B&K Precision Corporation, USA) at a constant voltage of 300 V, with other parameters selected based on our previous study^13^. The time-dependent relations of voltage during the processes were recorded directly and independently using free PC software for BK Precision MR Series power supplies. In contrast to the conventional MAO process, UMAO involves the utilization of a US wave generator (FSF-010 S, ChemLand, Poland) operating in two modes: sinusoidal wave or unipolar rectangular wave with a frequency of 40 kHz and a power of 80 W. Detailed characteristics of these waveforms are provided in the supporting information file for^13^. A comprehensive list of designated MAO and UMAO coatings formed with different parameters can be found in Table 2. For both processes, an aqueous electrolyte containing 14 g/L of calcium acetate hydrate Ca(CH_3_COO)_2_ (CA) (99%; Thermo Fisher Scientific, USA) and 3 g/L of β-glycerophosphate disodium salt pentahydrate C_3_H_7_Na_2_O_6_P·5H_2_O (β-GPNa) (≥ 97%; Merck KGaA, Germany) was employed as the source of calcium and phosphorus ions. Titanium specimens were utilized as the anode, while a platinum electrode served as the cathode. Each titanium disc was securely mounted onto a holder with a f12 mm O-ring. To dissipate the heat generated during the modifications, the system was situated in a water-cooling bath maintained below 20 °C. Following the treatment, the surfaces underwent a rinse with ultra-pure water and were subsequently dried in ambient air.

**Table 2:** The labels of MAO and UMAO coatings formed at different conditions.

Group	Label	Voltage [V]	Current intensity [mA] (current density [mA/cm^2^])	Time [s]	Ultrasound
−	Ti13Zr13Nb	−	−	−	−
68_450	68_450_n_300	300	68 (60)	450	−
	68_450_sin_300				sinusoidal
136_450	136_450_n_300		136 (120)		−
	136_450_rec_300				unipolar rectangular
136_600	136_600_n_300			600	−
	136_600_sin_300				sinusoidal
In the specimen labels, the first number refers to the current density (in mA/cm²), the second number indicates the processing time (in seconds), followed by the type of ultrasound application (“sin” for sinusoidal ultrasound, “rec” for rectangular ultrasound and “n” for MAO process – without ultrasound), and the last part denotes the applied voltage during the process (300 V for each specimen).					

### Characterization

#### Surface morphology analysis

The surfaces’ morphology of the uncoated and coated specimens was observed using a field emission scanning electron microscope (SEM, JEOL JSM-7800 F, JEOL Ltd., Japan) with image analysis performed using a secondary electron detector (SED) at 5 kV acceleration voltage.

#### Chemical and phase composition

The coatings’ elemental composition was identified through the X-ray energy-dispersive spectrometer (EDS) (Edax Inc., USA; 30 kV accelerating voltage) connected to the aforesaid SEM. The average values of each element for the tested specimen were determined from at least three repetitions of measurement at different spots on the specimen.

Phase analysis of coatings (*n* = 3) was studied by a Bruker^®^ X-ray diffractometer (D8 Advance, Karlsruhe, Germany), operating at 40 kV and 40 mA (2θ range between 15˚ and 90˚ with a step size of 0.05 and counting time 2 s). The analysis was performed working in Thin Film mode, with 3° of grazing angle. All the phases in the coatings were identified using the PDF2 database and the semi-quantitative analysis of the phase content was performed with High Score Plus software with the Rietveld method. The differences between measurements were minimal (below 0.2%), indicating the reliability of the obtained values.

#### Topography

Specimens’ topography (*n* = 3) was investigated by a 3D optical profilometer with a confocal technique (S neox 090, Sensofar, Spain) using a Nikon – EPI 20X objective with a magnification of 20x. Statistical parameters for the tested specimens in accordance with ISO 25,178 were analyzed based on conducted research using SensoMAP Standard 9 software. The measurements were conducted using the parameters: L-filter (λc) – Gaussian, 0.25 mm and S-filter (λs) – Gaussian, 2.5 μm.

#### Wettability and surface free energy

The wettability was evaluated using the water-falling drop method (drop volume of ~ 2 µL). The measurements were made using an optical tensiometer (Attention Theta Life, Biolin Scientific, Finland) and a dedicated OneTennsion program (Biolin Scientific, Finland) utilizing the Laplace–Young equation for computer image analysis. Each measurement had a duration of 10 s and was conducted at room temperature (about 20 °C). The contact angle (CA) of each specimen was specified for a polar solvent - demineralized water and a non-polar – diiodomethane (for synthesis, Merck KGaA, Germany). The surface free energy (SFE) of each specimen was determined using the Kaelble–Owens–Wendt method, which enables the calculation of the dispersion and polar components^23^. The mean values of the CA of each specimen were determined by averaging three measurements being made at different specimen locations, whereas SFE values were calculated from the average CA values.

#### Thickness measurements

To determine the thickness of the coatings, the specimens were sliced in half, embedded in epoxy resin, ground, and then polished. The cross-sections of the anodized specimens were examined for morphology using the SEM described above, with image analysis performed using a back-scattered electron detector (BSE) at an acceleration voltage of 5 kV. Five images were captured at various locations within the specimen cross-section, and five distinct points were measured within each photo. Subsequently, the average values of the 25 measurements were calculated.

#### Mechanical and Adhesive properties

The Anton Paar’s mechanical tester (Step 500 Surface Testing Platform, Anton Paar, Austria), equipped with the Micro Combi Tester (MCT³) and the Nano Hardness Tester (NHT³) heads, was used to conduct nanoindentation and scratch tests. The elastic modulus (E) and hardness (H) of the coatings were determined through 25 (5 × 5) indentation measurements on each specimen, with a 50 μm distance between indentations with Berkovich indenter. A maximum load of 20 mN, loading and unloading times of 20 and 15 s, respectively, as well as a 5 s dwell at maximum load, were applied. The Oliver-Pharr method^24^ was used to analyze load-penetration curves and obtain elastic modulus and hardness values. A Poisson coefficient of 0.3^25^ was assumed to convert the reduced elastic modulus to the elastic modulus (E) of the coatings.

Scratch tests were performed with a Rockwell C diamond stylus with a radius of 200 μm under a progressive load from 1 N to 20 N with a load rate of 9.5 N/min, and over a scratch length of 5 mm and a sample speed of 2.5 mm/min. An optical microscope was used to analyze the scratch path. The test assessed cohesive and adhesive damage of the coating by determining the critical loads Lc_1_ and Lc_2_ for initial cohesive cracks and complete perforation of the coatings leading to exposure of the substrate, respectively. These values were obtained by analyzing scratch test data and scratch paths by optical microscopy (US2, Olympus, Tokyo, Japan). Isolated damages were ignored^25,26^. The mean Lc_1_ and Lc_2_ values of each specimen were determined by taking three measurements on the specimen and then finding the mean. The distance between scratches was 1000 μm and the indenter was cleaned after each measurement.

### Cytocompatibility

The selected specimens were subjected to in vitro cytocompatibility experiments using a human osteoblast cell line (hFOB 1.19, RRID: CVCL3708; ATCC, USA). Reagents used, unless specified, were sourced from Merck KGaA (Darmstadt, Germany). The hFOB cells were cultured in a 1:1 mixture of Ham’s F12 Medium and Dulbecco’s Modified Eagle’s Medium (no phenol red, DMEM/F-12, Gibco™), supplemented with 0.3 mg/mL geneticin (G418) and 10% Fetal Bovine Serum (FBS) at 34 °C in a humidified atmosphere with 5% CO_2_. Subsequently, cells (8 × 10^4^) were seeded in a 100 µL drop directly onto the specimens (*n* = 4; surface area = 1.44 cm^2^). After adhesion (~ 4 h), a medium was added (up to 2 mL) to the wells. The experiments were carried out over 3 days. The MTT viability assay (3-(4,5-dimethylthiazol-2-yl)-2,5-diphenyltetrazolium bromide) was conducted following the procedures outlined in Protocol S2, as referenced in the supporting information of^13^.

### Corrosion examination

Corrosion tests were conducted using a potentiostat/galvanostat (Atlas 0531, Atlas Sollich, Poland) with a three-electrode system (*n* = 3). The analysis was carried out with AtlasCorr05 software to calculate corrosion current density and potential via Tafel extrapolation and polarization curves. The specimen was the working electrode, with a platinum rod as the counter electrode and a saturated calomel electrode (SCE) as the reference. The tests were conducted in 100 mL of Ringer’s solution (Merck KGaA, Darmstadt, Germany) at a temperature of 37 °C. Initially, open circuit potential (OCP) values were determined, followed by recording corrosion curves using the potentiodynamic method, ranging from − 1.0 V to + 1.0 V at a scan of 0.1 mV/s. Corrosion current density (j_corr_), zero current potential (E_j_=0), and polarization resistance (R_pol_) were determined using AtlasLab software (Atlas-Sollich, Poland).

### Statistics

Commercial software (SigmaPlot 15.0, Systat Software, San Jose, CA, USA) was utilized for statistical analysis of the data. The normal distribution of the data was assessed using the Shapiro-Wilk test. Results were reported as mean ± standard deviation (SD) and analyzed statistically using one-way analysis of variance (one-way ANOVA). Multiple comparisons with the control group were conducted using the Bonferroni t-test, with statistical significance considered at *p* < 0.05.

## Results and discussion

### Coatings’ formation

**Fig. 2:**
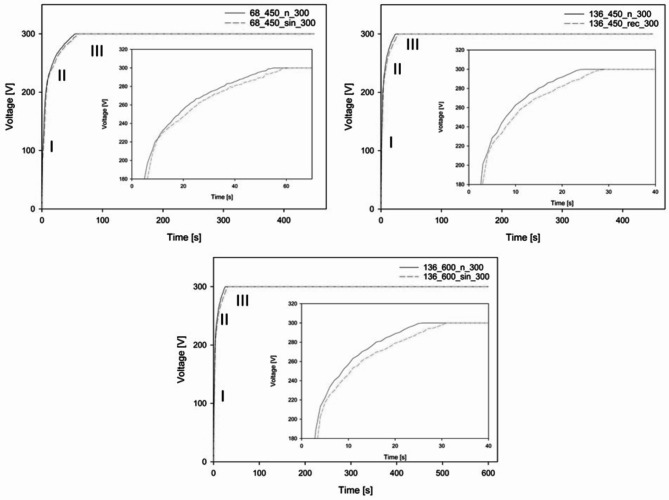
Change in voltage vs. treatment time for the MAO and UMAO processes at different conditions. The characteristics of the curves can be divided into three typical stages.

The characterizations of the MAO and UMAO processes were examined based on the time-dependent relationship between voltage, as illustrated in Fig. 2. The subgraphs in the individual plots represent enlarged images showing the voltage differences between the MAO and UMAO specimens at voltages from 180 to 300 V at the respective time ranges. Both processes have characteristic curves that can be divided into three main stages^25,27^. The voltage increases rapidly in the I stage until the characteristic breakdown voltage is obtained, which is defined as the lowest voltage that can destroy the oxide coating formed on the substrate. The breakdown voltage is lower in all groups for the UMAO compared to the MAO process, consistent with the results obtained by Xu et al.^17^. The reduction in the breakdown voltage is caused by bubbles generation in the electrolyte due to US. The constantly repeated, US cavitation causes these bubbles to grow, and oscillations generated by the US make them contract and eventually burst. This phenomenon generates a microenvironment on the specimen surface characterized by high temperature and/or high pressure, supplying extra energy and lowering the voltage^17,19,28^.

### Morphology and chemical composition’s observations

**Fig. 3:**
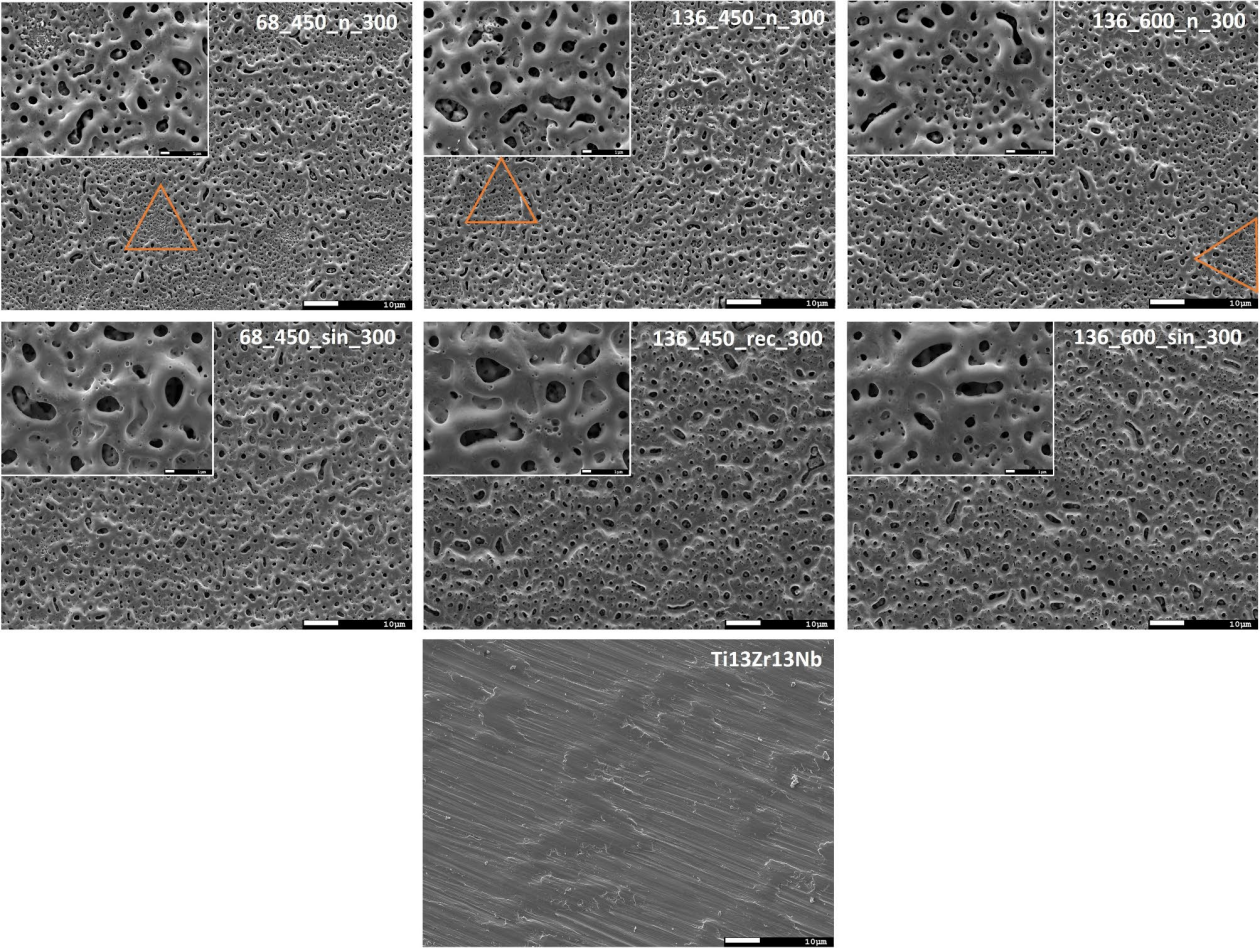
SEM images of uncoated and coated Ti13Zr13Nb specimens at 500× (larger image) and 5000x (smaller image) magnifications. Porous coatings were obtained, with their structure being influenced by the process parameters. The orange triangle indicates areas with a concentration of nanopores.

SEM studies were conducted to analyze the morphology of the coatings. The MAO process caused notable changes in the surface morphology of the Ti13Nb13Zr alloy specimens. Both MAO and UMAO coatings (Fig. 3) exhibited porous, rough, and highly folded characteristics, consistent with previous studies^16,17,22,25,29,30^. In contrast to UMAO, MAO coatings display regions with visible nanopores concentrations, especially in specimen 68_450_n_300. Similar nanopore regions were noticed for MAO coatings obtained in sodium phosphate tribasic dodecahydrate electrolyte on the Ti18Zr15Nb titanium alloy by Farrakhov et al.^31^. However, the size and distribution of pores on oxidized alloys may vary based on the substrate structure^32^. For instance, nanotubes generated on the α phase of the Ti6Al4V alloy through anodic oxidation had thinner walls and larger internal diameters compared to those on the β phase^33^. The Ti13Zr13Nb alloy exhibits a microstructure with α, α’, and β phases^34^; however, no distinct morphologies of the coatings were observed within the sample. Interestingly, UMAO coatings were more homogeneous than MAO coatings. This uniformity may be the US effect, which increases the electrolyte flow rate and simultaneously leads to more even heat distribution on the specimen surface^17^.

According to the MAO process theory, the elements present in the electrolyte are incorporated into the coating generated on the modified substrate^25^. This was confirmed in all analyzed specimens, indicating the process was performed adequately. The effects of US (and its mode) or process parameters on the chemical composition of coatings were minimal (see Table S1) since Ti ranged from 49.09 to 50.62 wt%, Zr from 7.75 to 8.06 wt%, Nb from 7.67 to 8.13 wt%, P from 2.19 to 2.76 wt%, Ca from 1.42 to 1.64 wt% and O from 29.70 to 31.31 wt%. These findings contradict Xu et al.^17^, who noted an increase in oxygen concentration in coatings on Ti6Al4V specimens treated with the MAO using the US. Conversely, Kazantseva et al.^18^ did not observe any effect of the US on the chemical composition of CaP coatings on commercially pure titanium. It is essential to consider that the MAO process is affected by various factors, and the chemical composition of the coatings may be influenced by the substrate material and process parameters, which differed between our study and the ones mentioned^6^. Moreover, the penetration depth of the electron beam in EDS is influenced by factors such as the incidence energy (applied voltage) and substrate material, which can affect the accuracy of elemental analysis^35^. Variations in these factors between studies can lead to differences in reported chemical compositions. Thus, while our findings are consistent with some studies, they may differ from others due to these inherent limitations in EDS analysis.

### Surface topography

**Fig. 4:**
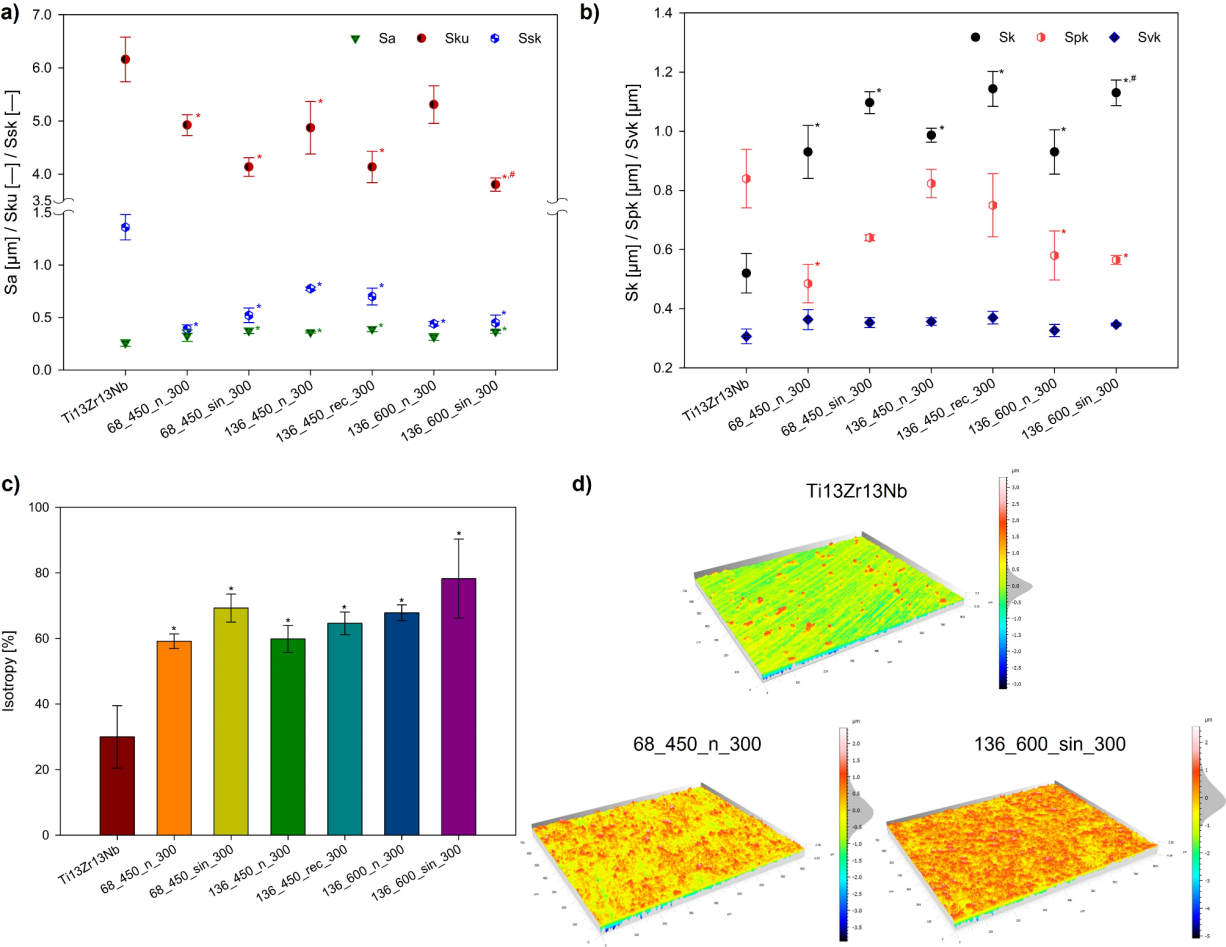
(**a**) The average values of the heigh parameters: kurtosis (Sku), and skewness (Ssk) for uncoated and coated specimens. (**b**) The average values of the functional parameters: core height (Sk), reduced peak height (Spk), and reduced valley depth (Svk) for uncoated and coated specimens. (**c**) The calculated surface texture direction values showed mixed structure for all modified specimens. (**d**) Surface topographies and the histogram of peak and valley distribution of uncoated Ti13Zr13Nb, 68_450_n_300 – specimen after treatment with the lowest isotropy value, 136_600_sin_300 – specimen after treatment with the highest isotropy value. For all analyses *n* = 3; data are expressed as means ± SD; * statistically significant difference as compared to the Ti13Zr13Nb (*p* < 0.05). # – statistically significant difference as compared to the MAO specimen in each group (*p* < 0.05).

The surface topography of the specimens was quantitatively described by analyzing the average values of heigh parameters (Fig. 4a): roughness (Sa), kurtosis (Sku), skewness (Ssk), and functional parameters (Fig. 4b): core height (Sk), reduced peak height (Spk), reduced valley depth (Svk). Furthermore, the isotropy (Is) of all specimens was analyzed (Fig. 4c). The roughness of all coated specimens is similar, ranging from ~ 0.31 μm to ~ 0.39 μm for 136_600_n_300 and 136_450_rec_300 specimens, respectively. The US, whether sinusoidal or unipolar rectangular, increased this parameter compared to conventional MAO samples, aligning with other research findings^36^. Despite the unipolar rectangular ultrasound having a longer maximum amplitude and therefore causing a more intense process than sinusoidal ultrasound, it did not produce rougher surfaces^37^. The MAO process parameters likely had a more dominant influence on the surface characteristics. The Sku values for all coatings were above 3.0, and the US decreased these values, indicating a more condensed distribution compared to the normal distribution. These Sku values are represented as a “bell” shape (Fig. 4d) and are identified as platykurtic^38^. The distribution of peaks and valleys was symmetrical in MAO specimens (Fig. 4d for 68_450_n_300), indicating an even surface distribution. UMAO specimens showed an increase in the number of valleys, evidenced by a histogram spectrum in the − 0.3−-0.2 μm range (Fig. 4d for 136_600_sin_300). The skewness measures the asymmetry of the height distribution and serves as a valuable parameter for peaks or valleys anticipation, illustrating the relationship between their occurrence^39,40^. Positive Ssk values indicate a prevalence of peaks in the surface^41^. Our previous study^13^ on commercially pure titanium did not find a distinct effect of ultrasound type on Ssk value. However, in this study on Ti13Zr13Nb, sinusoidal ultrasound increased Ssk, while unipolar rectangular ultrasound decreased it. The most pronounced asymmetry was in specimen 136_450_n_300, while the least was in 68_450_n_300. Nonetheless, low Ssk values suggest a reduced probability of crack nucleation^42^.

Functional parameters in the Sk group assess the tribological properties of materials^43,44^. In bone tissue engineering, Sk measures the core roughness where the load is dispersed after biomaterial implantation^13^. A higher value is desirable as it indicates better load distribution. The US increased Sk by ~ 15–20%, depending on the group, with no significant differences observed between the various modes of ultrasound. Further, Spk and Svk are closely connected to the Sk. A smaller Spk value is favorable as it indicates less coating removal during initial contact with surrounding tissue^13^. Excluding the 68_450 group, ultrasound reduced the value of Sk, with the unipolar rectangular ultrasound showing a significant decrease, suggesting improved wear resistance compared to MAO coatings. Svk quantifies the depth of valleys beneath the core roughness, which can facilitate cell growth and long-term implant integration^45–47^. The Svk values for MAO and UMAO coatings were comparable, ranging from ~ 0.32 μm to ~ 0.37 μm, indicating similar lubricant retention and favorable environments for cell proliferation^42,48^.

Biomaterial coatings must be uniformly distributed to prevent weak spots that compromise durability and corrosion resistance in body fluids^12,15,49^. Therefore, examining surface isotropy is crucial^50^. MAO and UMAO specimens exhibited a mixed structure (20% ≤ Is ≤ 80%)^51^ and the US consistently improved isotropy across all groups, with sinusoidal ultrasound having a more favorable effect on the isotropy of the coatings compared to unipolar rectangular ultrasound. The highest isotropy value (~ 78.28%) was for 136_600_sin_300, while the lowest (~ 59.19%) for 68_450_n_300. Previous studies^13^ showed that the US can affect surface anisotropy depending on the MAO process variables. Differences in substrate isotropy, despite similar treatment, indicate the process effect and substrate influence^6^. The US enhanced surface anisotropy, resulting in uniform surface structure parameters in all directions. Nevertheless, isotropy levels ideally should exceed 80%^13,51^.

### Contact angle and surface free energy

Table 3 lists the contact angles for water (polar liquid) and diiodomethane (non-polar liquid), as well as the calculated polar and dispersive surface free energy components for all specimens. The sum of these components determines the total surface free energy^23^. The analysis revealed that the US decreases the wettability of non-polar liquids while enhancing the polar component’s wettability of the coatings. The water contact angle values for the modified specimens ranged from ~ 43.95° to ~ 70.17°. These values align with those reported by Dziaduszewska et al. for CaP-based MAO coatings on Ti13Zr13Nb titanium alloy, which ranged from ~ 35.63° to ~ 64.82°, depending on the MAO process parameters^25^. Generally, surface wettability is vital for the quality of biomaterials, particularly in bone implants. Moderate hydrophilicity improves cell adhesion, proliferation, and differentiation while hindering bacterial adhesion and potentially preventing inflammation^1^. Optimal contact angle values for biomaterials range from 35 to 80°, enhancing cell proliferation and behavior^6,52^. All modified specimens in this study comply with this requirement.

Determining surface free energy is crucial as it dictates the adhesion of the implant to the body fluids^53^. The higher surface energy of a solid object increases its likelihood of interaction with other liquids^54^. When proteins or cells approach the implant, their surface domains align to minimize the overall free energy^55,56^. The US increased the dispersion component of SFE in each studied group. Notably, it decreased the polar component, resulting in higher values of the water contact angle. These findings are in line with expectations, as the primary factor contributing to water’s surface tension is the polar component^54^. The highest value of SFE was observed for specimen 136_450_rec_300, which can be correlated with the highest roughness. Nevertheless, both surface wettability and SFE are influenced by the specimen’s morphology, chemical composition, and topography^1,6^. Establishing a correlation between these parameters is immensely challenging^53,57^.

**Table 3.**
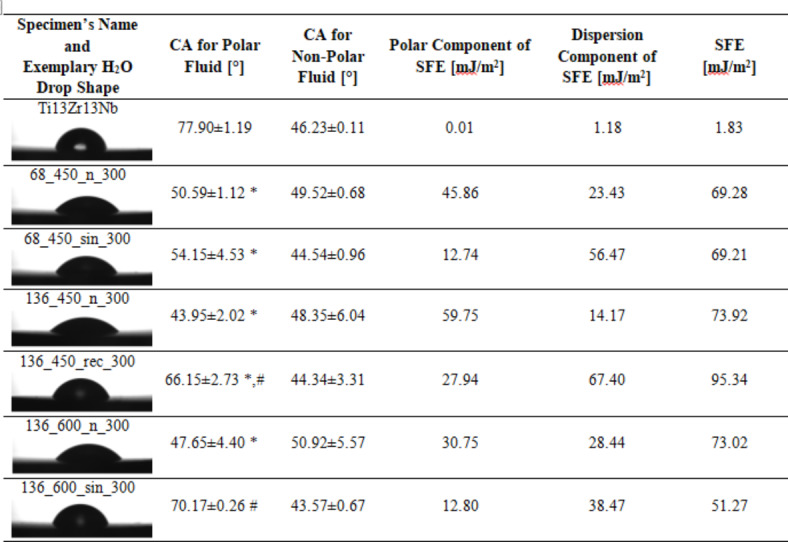
Contact angles and surface free energies of all specimens.

CA – contact angle; SFE – surface free energy. For CA analyses *n* = 3; data are expressed as means ± SD; * statistically significant difference as compared to the Ti13Zr13Nb (*p* < 0.05). # – statistically significant difference as compared to the MAO specimen in each group (*p* < 0.05). There is no statistically detected difference between samples in the groups for CA for non-polar fluid assays in our study.

### Thickness

**Fig. 5:**
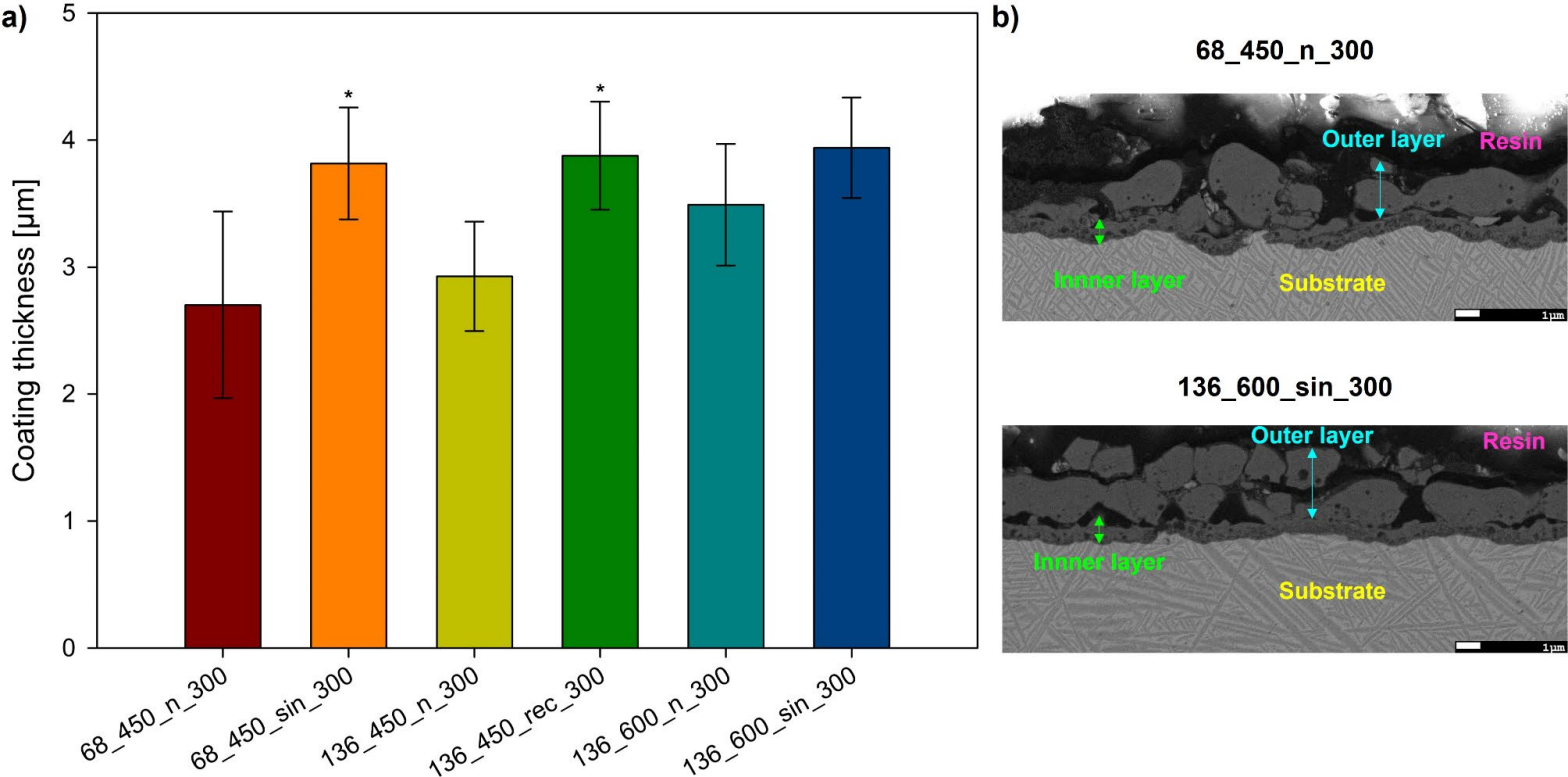
(**a**) Thickness values of the MAO and UMAO coatings generated under different conditions (*n* = 20). The use of ultrasound during the MAO process increased the thickness of the coatings. All data are expressed as means ± SD; * ─ statistically significant difference as compared to the MAO specimen in each group (*p* < 0.05). (**b**) Cross-section SEM micrographs of coatings of 68_450_n_300 – specimen after treatment with the lowest coating’s thickness value and 136_600_sin_300 – specimens after treatment with the highest coating’s thickness value.

Figure 5a shows the thickness values of the MAO and UMAO coatings, and Fig. 5b illustrates the morphology of selected cross-sections of the specimens. The MAO coating thickness ranged from ~ 2.7 – ~3.5 μm, while the UMAO from ~ 3.8 – ~3.9 μm, depending on the process parameters. All coatings consisted of a dense inner layer and a more porous outer layer, with a distinct boundary between them. The US resulted in thicker coatings across all tested groups, consistent with previous studies on commercially pure titanium and aluminum alloy^13,19^. This increase in thickness can be attributed to the additional energy and better mass transfer facilitated by the US^28^. Additionally, coatings produced via the UMAO exhibit improved compactness and uniformity compared to the MAO process (Fig. 5b). The interface between the substrate and internal layer, as well as the external layer and resin, appeared more uniform and straight in the UMAO specimen. This may be due to the effects of the US, which enhance electrolyte flow rate and ensure more even heat distribution, promoting uniform coating growth^17^.

### Phase composition

**Fig. 6:**
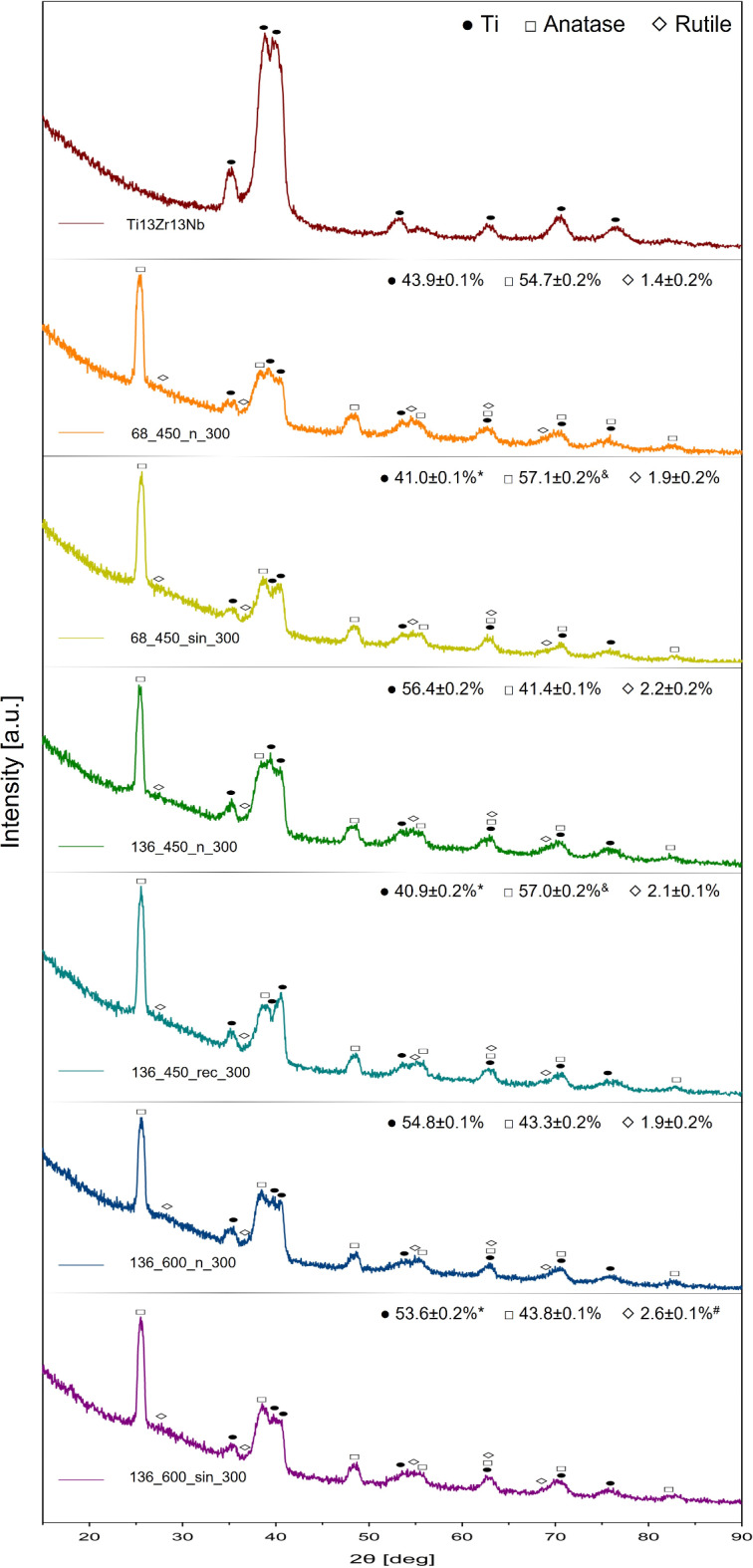
XRD analyses for all specimens (representative of three analyses for each modified specimen) and the volume fractions of anatase and rutile phases in modified specimens (semi-quantitative analysis performed with the Rietveld method; *n* = 3). In all modified specimens, anatase peaks are more visible than rutile peaks. Ultrasound application enhanced the crystallinity of the coatings. *, &, # ─ statistically significant differences as compared to the MAO specimen in each group for Ti, anatase and rutile, respectively (*p* < 0.05).

The XRD analysis shows the peaks from the titanium substrate on all the specimens due to the low thickness of the MAO/UMAO coatings. In untreated specimens, only these peaks are visible, indicating no modifications. In all treated specimens, peaks of TiO_2_ in both rutile and anatase forms are present (Fig. 6), indicating a strong oxidation reaction during the processes^58^. The sinusoidal US simultaneously increased the volume of both the anatase and rutile phases, while the rectangular US significantly increased the anatase (Fig. 6). In all modified specimens, anatase peaks are more prominent than rutile peaks, confirmed by semi-quantitative analysis showing a higher quantity of anatase compared to rutile (Fig. 6). Specifically, the quantity of anatase ranges from 41.4 to 57.1% (for 136_600_n_300 and 136_450_rec_300, respectively), whereas rutile ranges from 1.4 to 2.6% (for 68_450_n_300 and 136_450_n_300, respectively). Overall, sinusoidal US increases the volume of crystalline phases by ~ 5%, while rectangular US increases it by over ~ 25%. The results are consistent with the literature, where Ajiriyanto et al.^59^ and Xu et al.^17^ confirmed that the US increased the quantity of crystalline phases in MAO coatings on zircaloy-4 and Ti–6Al–4 V alloy, respectively. However, Wu et al.^12^ did not observe that US affects the phase composition of MAO coatings, suggesting that the power and frequency of US might not have been sufficient for lattice distortion during the transition process^12^. Variations observed in this study for different types of USs are noticeable. The unipolar US maintains a maximum amplitude longer than the sinusoidal US, contributing to changes in composition due to higher energy transfer^13,37^. Furthermore, no additional diffraction peaks were detected, even though EDS analysis revealed the incorporation of Ca and P ions (Table S1). This suggests that additional phases might exist in the amorphous or crystalline structure below the XRD detection limit^25^. These findings are consistent with Zhang et al.^60^, who did not observe any hydroxyapatite peaks in the MAO coatings on pure titanium using a solution of calcium acetate monohydrate and β-glycerophosphoric acid disodium salt pentahydrate.

### Mechanical properties

**Fig. 7:**
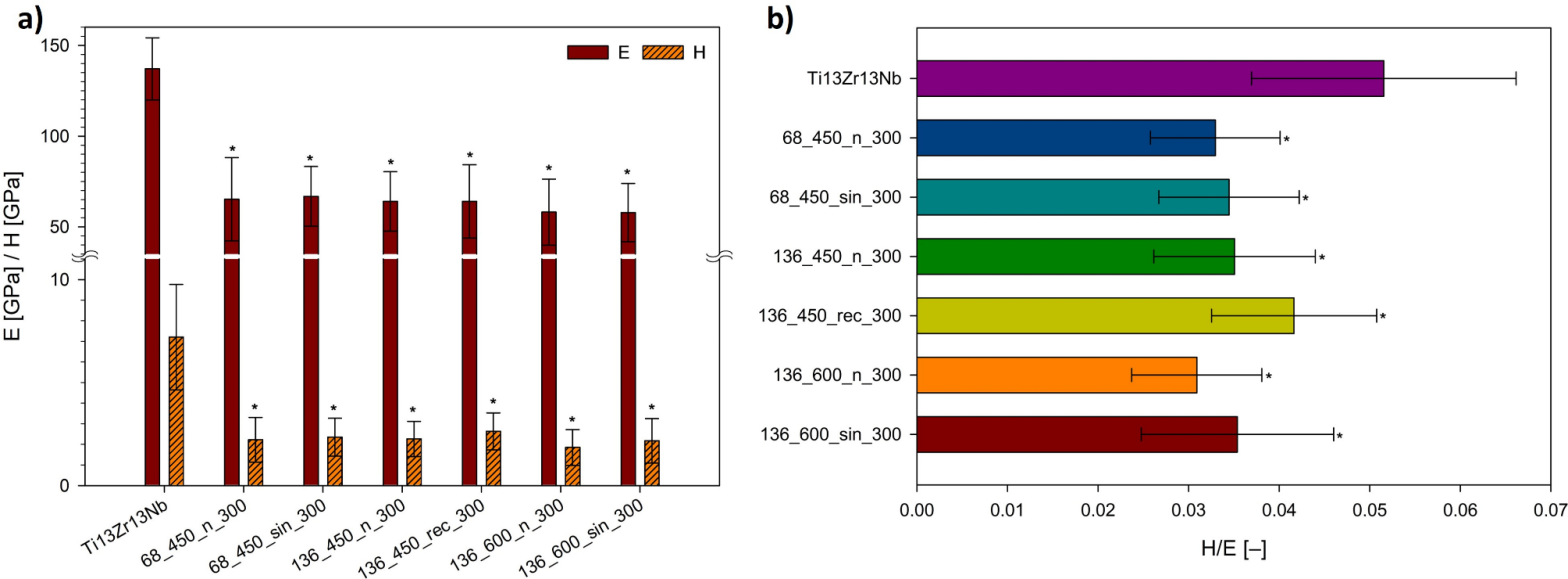
Nanomechanical properties of MAO and UMAO coatings: (**a**) elastic modulus (E) and hardness (H), and (**b**) H/E ratio values. The coatings show lower values of elastic modulus and hardness compared to the uncoated specimen. All data are expressed as means ± SD, *n* = 25; * ─ statistically significant difference as compared to the uncoated Ti13Zr13Nb (*p* < 0.05).

The nanomechanical properties of the coatings, including elastic modulus (E), hardness (H), and H/E ratio (Fig. 7), are critical for determining the performance and longevity of implants. These properties are essential for ensuring that the mechanical characteristics of the coatings match those of the replaced bone, minimizing stress shielding and implant loosening^6,13,25^. Among the modified specimens, the highest E was observed in 68_450_sin_300 (66.8 ± 16.4 GPa), while the highest H was noted in 136_450_rec_300 (2.6 ± 0.9 GPa). Conversely, the lowest E was recorded for 136_600_sin_300 (57.9 ± 16.1 GPa), and the lowest H was found in 136_600_n_300 (1.8 ± 0.9 GPa). All MAO and UMAO modifications affect the mechanical properties of the biomaterial, which has an elastic modulus of ~ 137.0 GPa and hardness of ~ 7.2 GPa. These modifications relate to the formation of a coating on the substrate, composed of complex oxides which hardness can vary depending on the phases present, among other factors^6,25^. Notably, the hardness of UMAO coatings increased with the application of US, along with surface roughness. Ultrasound improves MAO coating formation by boosting mass transfer and energy input, which could lead to thicker, more uniform coatings with increased hardness due to additional nucleation sites, altered microstructure, and the formation of harder rutile phases^13,25,37,61^. On the other hand, the US did not relevantly influence elastic modulus values within groups, but the standard deviations were smaller for the coatings treated with the sinusoidal US, correlating with the isotropy of the coatings. Furthermore, the use of US increased the hardness, for example, by almost ~ 18% for the 136_600 group. These observations align with the findings by Guo et al.^62^, who reported that the UMAO process on 6063 aluminum substrates increased the hardness of the coatings and Ajiriyanto et al.^59^, who indicated the same trend for coatings on zircaloy-4. The highest H/E value for the modified specimen was observed in the 136_450_rec_300 specimen (0.042 ± 0.09), while the lowest was in the 136_600_n_300 specimen (0.031 ± 0.07). According to the literature, a high H/E ratio (greater than 0.1) indicates high wear resistance^25^. It is noteworthy that the US consistently increased this parameter, bringing it closer to the desired value of 0.1. The best fracture toughness was attributed to the UMAO coating generated at 136 mA and 450 s with unipolar rectangular US. However, it is important to mention that this indicator may not account for the influence of plasticity on fracture toughness. Although plastic deformation is minimal and elasticity primarily governs fracture toughness in ceramic coatings, this parameter should be considered an approximation^63^.

### Adhesive properties

**Fig. 8:**
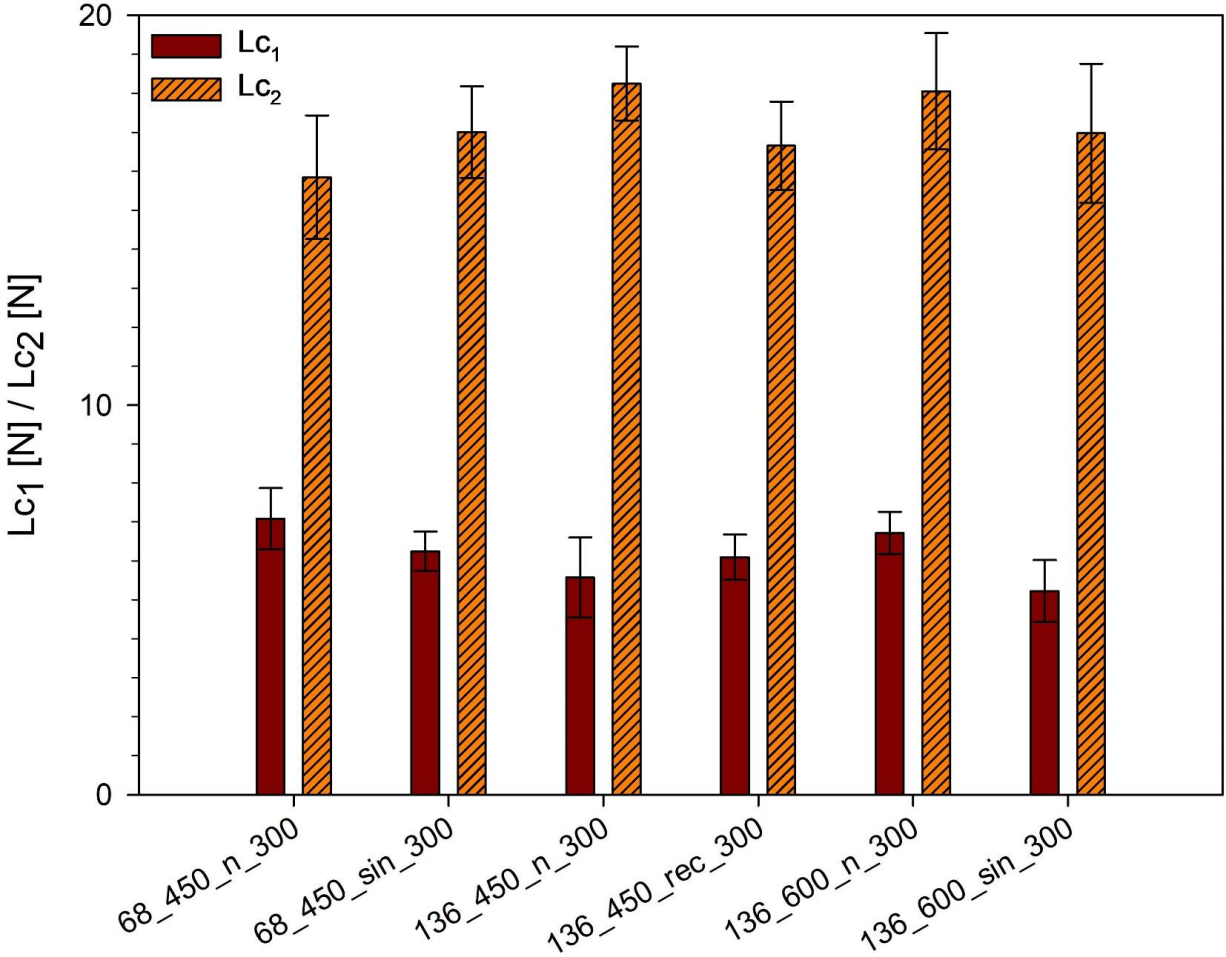
Load results in initial cohesive cracks (Lc_1_) and critical load causes total damage of the coating (Lc_2_) values for the MAO and UMAO coatings (*n* = 3). All samples experience coating delamination, but the force at which the substrate becomes visible varies. All data are expressed as means ± SD. There is no statistically detected difference as compared to the MAO specimen in each group.

Scratch test is a technique commonly used to determine the adhesion values of the MAO coatings described by critical load^25,64^. These values are obtained by analyzing the load of the indenter, penetration depth, friction force, and coefficient, as well as acoustic emission signal and inspecting the scratch path^13,65^. Although all specimens experience coating abrasion, the substrate’s exposure point varies (Description, Figure S1). The highest load results in initial cohesive cracks (Lc_1_) were observed for specimen 68_450_n_300, and the highest critical load causes total damage of the coating (Lc_2_) was noted for 136_450_n_300 (Fig. 8). Interestingly, the US application beneficially affects the adhesion only for the 68_450_n_300 group, while in the 136_450 and 136_600 groups, it resulted in coating failure at narrowly lower forces (a decrease of ~ 8% and ~ 6%, respectively). Our recent research^11^ found that the US can either increase or decrease the coating adhesion on commercially pure titanium, depending on the MAO process parameters and the US types. This study on Ti13Zr13Nb titanium alloy showed no clear relationship. It is widely acknowledged MAO is an effective method for enhancing coating adhesion compared to other surface modification techniques; nevertheless, the coating often exhibits inherent brittle behavior^13,20,25,65^. Moreover, MAO process parameters significantly affect coating adhesion to the substrate^6,25,65^. Using the US can facilitate the process, potentially leading to more intense discharges on the metallic substrates and improving the scratch resistance of the coatings^66^. However, nonlinear effects induced by the US may introduce variability in this appearance^46^. Therefore, optimizing these parameters and the US mode can enhance the adhesive properties of ceramics coatings, including those produced via US-assisted MAO.

### Influence of ultrasound on coating’s properties concerning potential biomedical application

The MAO method is relatively novel, with the coating formation mechanism not yet thoroughly studied. Due to the numerous variable parameters of the process (such as time, current density, etc.) and the potential for modification through additional techniques (e.g., US), identifying the optimal combinations for developing coatings suitable for biomedical applications is highly complex^13^. In our work, we focused on coatings intended for implantology, specifically using calcium phosphates. Selecting an appropriate CaP coating for biometals like titanium alloy involves a comprehensive evaluation of their properties^6^. Several experiments were conducted to determine the impact of US on the properties of the MAO/UMAO coatings (the processes were conducted with varying parameters, including different times and current values, to verify the schematics of US effects), with a summary of these findings in relation to the requirements for modern biofunctional coatings presented in Table 4. The results indicate that varying MAO process parameters and different US modes significantly affect the physicochemical and mechanical properties of the coatings, which is profoundly discussed in the sections above. Concerning implantology applications, using the sinusoidal US in the 136_600 group notably boosted height and functional topography parameters, isotropy, thickness, and mechanical properties of the coatings (as indicated by green arrows). In contrast, some of these properties were not ameliorated in other groups (as indicated by red arrows). Based on the findings presented in Table 4, the use of the US enhanced most or all the properties of the MAO coating in the discussed aspect regarding biomedical applications; therefore, UMAO specimens were selected for further research.

**Table 4:** The influence of ultrasound (sinusoidal for 68_450 and 136_600 groups and unipolar rectangular for 136_450 group) on coatings properties concerning biomedical applications across various groups.

Group	Coatings properties													
	Sa [µm]	Ssk [–]	Sk [µm]	Spk [µm]	Svk [µm]	Is [%]	CA-*P* [°]	CA-NP [°]	d [µm]	c [%]	H [GPa]	E [GPa]	H/E [–]	Lc_2_ [*N*]
**68_450**	**↑**	**↑+**	**↑**	**↑**	**↓**	**↑**	**↑+**	**↓**	**↑**	**↑**	**↑**	**↑**	**↑**	**↑**
**136_450**	**↑**	**↓**	**↑+**	**↓**	**↑+**	**↑**	**↑+**	**↓**	**↑**	**↑**	**↑**	**↑**	**↑+**	**↓**
**136_600**	**↑**	**↑**	**↑**	**↓+**	**↑**	**↑+**	**↑+**	**↓**	**↑+**	**↑+**	**↑**	**↓+**	**↑**	**↓**
Sa – roughness; Ssk – skewness; Sk – core height; Spk – reduced peak height; Svk – reduced valley depth; Is – isotropy; CA-P– contact angle for polar fluid (water); CA-NP – contact angle for non-polar fluid (diiodomethane); d – thickness; c – crystallinity; E – elastic modulus; H – hardness; H/E – hardness to elastic modulus ratio; F_c_ – complete delamination force. Legend: green arrow – positive influence of US to the coatings’ feature for biomedical application, red arrow – negative influence of US to the coatings’ feature for biomedical application, + – the most favorable result of the assessed quality for biomedical application (Ssk, Sk, Svk, Is, d, H/E: the highest; Spk: the lowest; CA-P: in the range of 35–80°; c: the highest quantity of rutile; E: the closer to 10–40 GPa).														

### Cytocompatibility

**Fig. 9:**
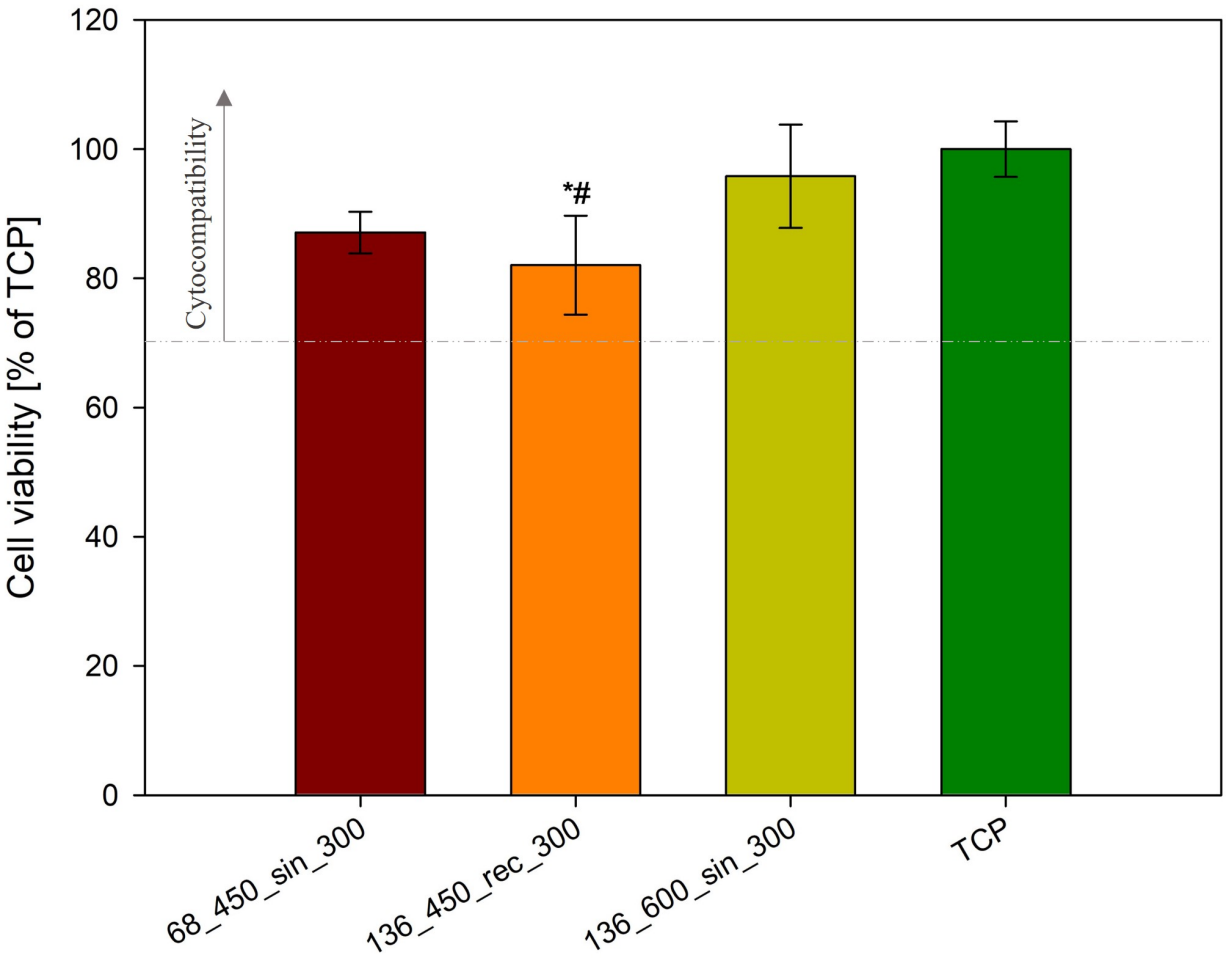
fHOB viability on tested specimens after 3-day culture. Results are expressed as a percentage of cell viability compared to the cell viability on the tissue culture plate (TCP) (*n* = 4; data are expressed as the mean ± SD; * statistically significant difference as compared to TCP (*p* < 0.05); # statistically significant difference as compared to the 136_600_sin_300 specimens (*p* < 0.05); there is no statistically detected difference between other specimens). All coatings are cytocompatible.

UMAO exhibited ameliorated coating properties suitable for biomedical applications. Consequently, these specimens underwent cytocompatibility tests with the hFOB 1.19 osteoblast cell line and the direct method of seeding cells onto the coating^67^. The application of various process parameters during the UMAO process influenced the cytocompatibility of the coatings. All specimens can be classified as cytocompatible, as indicated by cell viability well above the required threshold of 70%^68^ (Fig. 9). Specifically, specimens 136_600_sin_300 exhibited hFOB cell viability after 72 h of culture that closely resembled standard conditions (100% on tissue culture plate (TCP)) at ~ 96%. It is widely recognized that osteoblast viability depends on numerous factors, including the morphology, chemical composition, topography, crystallinity, wettability, and surface energy of the coatings^6^. For example, cytotoxicity studies by Xu et al.^69^ on pure titanium revealed that adding silicon to the sodium phosphate MAO electrolyte reduced the viability of neonatal rat calvarial cells, despite silicon being regarded as a bioactive material that can enhance the adhesion, proliferation, and differentiation of osteoblast-like cells. Furthermore, Xu et al.^17^ investigated the impact of US on the properties of MAO coatings on Ti6Al4V titanium alloy and found that the number of human osteoblast-like cells (MG-63) was higher for the UMAO specimen compared to the MAO specimen. The authors indicate that the US could have a beneficial influence on the morphology and topography of the coatings, leading to a higher proliferation rate. In our recent research^13^ on commercially pure titanium, we found that incorporating US during the MAO process significantly increased the regularity of the morphology and the pore size of the coatings, which profitably influenced the adhesion and proliferation of the human osteoblast cell line (hFOB). These results are consistent with our observations, where the highest cell viability was found in specimen 136_600_sin_300, which also exhibited the highest isotropy. Furthermore, the coatings of this specimen showed the highest quantity of rutile and the lowest elastic modulus, which could contribute to enhanced cell proliferation^6^.

### Corrosion properties

Based on the conducted research, specimen 136_600_sin_300 was identified as the most promising for biomedical applications, exhibiting superior biocompatibility alongside optimal physicochemical and mechanical characteristics. As biomaterials must demonstrate high corrosion resistance in human body fluids to ensure long-term functionality and biocompatibility^6^, the corrosion properties of this specimen were tested in Ringer’s solution - a standard simulated body fluid commonly to replicate physiological conditions, underscoring the coating’s suitability for biomedical use^70,71^. The corrosion results, shown in Table 4 and Figure S2, revealed an increase in the open circuit potential (OCP) of the modified specimen compared to the Ti13Zr13Nb alloy, with the OCP stabilizing at more noble potentials, suggesting that the coating’s barrier effect remained intact after 1 h of immersion^72^. However, the OCP values differed from the zero current potential determined by Tafel extrapolation, rendering them unreliable as a measure of corrosion tendency in this study. Potentiodynamic polarization analysis demonstrated active-to-passive transitions in both coated and uncoated specimens. Surface modification resulted in a slight increase in corrosion current density (~ 17%) and a decrease in polarization resistance (~ 4%), impacting overall corrosion protection. These findings align with some studies^13,70^, indicating that surface modifications often enhance biological properties but can reduce corrosion resistance due to imperfections like microcracks and potential gradients within the coating, leading to the formation of electrochemical “top-surface to pore-bottom” cells^70^. Conversely, several studies^11,30,73^ suggest that MAO can improve corrosion resistance, particularly when low current densities are applied over extended periods, reducing porosity and increasing coating thickness. Despite these concerns, the polarization resistance of the modified specimen remained significantly high (~ 542 kΩ·cm²), comparable to or exceeding the values reported in another study^71^ involving the Ti13Zr13Nb alloy and the MAO process, where polarization resistance ranged from 457.8 to 490.6 kΩ·cm² for modified specimens.

**Table 5:** Results of corrosion examinations (*n* = 3): open circuit potential (OCP), zero current potential (E_j=0_), corrosion current density (j_corr_) and polarization resistance (R_pol_) values.

Specimen	OCP [mV]	E_j=0_ [mV]	j_corr_ [nA/cm^2^]	*R*_pol_ [kΩ⋅cm^2^]
Ti13Zr13Nb	–129 ± 39	–241 ± 19	126 ± 32	562 ± 63
136_600_sin_300	227 ± 51*	–395 ± 4*	147 ± 10	542 ± 33

## Conclusions

In the present study, we successfully generated both micro-arc oxidized and ultrasound micro-arc oxidized coatings on Ti13Zr13Nb titanium alloy dedicated to biomedical applications. The MAO process in calcium phosphate-based electrolyte resulted in a porous surface morphology, while the US application led to greater homogeneity of these surfaces. Regardless of the MAO process parameters, the use of US increased the surface roughness, core height, isotropy, thickness, and contact angle for polar fluid, as well as the hardness-to-elasticity ratio of coatings. Moreover, from a biomedical perspective, the application of US positively impacted the coating by slightly increasing the quantity of rutile phase, skewness, and reduced peak height parameters, while lowering the elastic modulus for specimens processed under the following parameters: 600 s, 136 mA, 300 V, with sinusoidal ultrasound. The MTT assay using the human osteoblasts confirmed that all UMAO specimens were cytocompatible, with the highest cell viability observed for the 136_600_sin_300 specimen. This excellent osteo-compatibility could be attributed to the highest isotropy, the lowest elastic modulus and the greatest quantity of rutile in the coating, although other factors, such as coating thickness and wettability, should also be considered. Future research should include a quantitative analysis of surface topography to better understand the influence of the US on the homogeneity of MAO coatings, detailed EDS mapping to accurately assess the elemental distribution across the coatings, electrochemical impedance spectroscopy to highlight the differences between the MAO and UMAO conditions and focus on long-term in vivo studies to thoroughly evaluate the performance and longevity of UMAO coatings. Additionally, exploring bacterial adhesion on these surfaces would provide valuable insights into their potential effectiveness in preventing infections associated with medical implants. In conclusion, the integration of ultrasound in the MAO process significantly enhances coating properties, making them highly suitable for biomedical applications, particularly in implantology, such as hip or knee replacements. Our findings suggest a promising avenue for improving the effectiveness and durability of medical implants, thereby advancing patient care and treatment outcomes.

## Electronic supplementary material

Below is the link to the electronic supplementary material.

Supplementary Material

Supplementary Material - Figure S1

Supplementary Material - Figure S2

## Data Availability

Data sets generated during the current study are available from the corresponding author on reasonable request.

## Supporting information

The surface chemical composition (*n* = 3) (by weight%) of coatings determined using EDS analysis (Table S1); analysis and discussion of the results obtained in scratch studies (Description S1); the scratch path and the relationship of frictional force, penetration depth, and scratch distance on normal force with the indicated critical loads (Lc_1_ and Lc_2_) of the 68_450_n_300 and 136_450_rec specimens (Figure S1); open-circuit potential vs. time and potentiodynamic polarization curves recorded for uncoated Ti13Zr13Nb and 136_600_sin_300 specimens (Figure S2) – PDF.
