# Supplementary material for: Effect of ultrasound on the physicochemical, mechanical and adhesive properties of micro-arc oxidized coatings on Ti13Nb13Zr bio-alloy
Source: Sci Rep. 2024 Oct 25;14:25421. doi: 10.1038/s41598-024-75626-4 (PMC11511822; doi:10.1038/s41598-024-75626-4)
Supplement: Supplementary file 1 — Supplementary Material [file 41598_2024_75626_MOESM1_ESM.docx]

**Supporting Information for**

**Effect of Ultrasound on the Physicochemical, Mechanical and Adhesive Properties of Micro-Arc Oxidized Coatings on Ti13Nb13Zr Bio-Alloy**

Balbina Makurat-Kasprolewicz ^1,2,*^, Marcin Wekwejt ^3^, Luca Pezzato ^4^, Anna Ronowska ^5^, Jolanta Krupa ^6^, Sławomir Zimowski ^6^, Stefan Dzionk ^7^, Agnieszka Ossowska ^1^

^1^ Department of Materials Science and Technology, Gdańsk University of Technology, 80-233 Gdańsk, Poland

^2^ Department of Machine Design and Medical Engineering, Gdańsk University of Technology, 80-233 Gdańsk, Poland

^3^ Department of Biomaterials Technology, Gdańsk University of Technology, 80-233 Gdańsk, Poland

^4^ Department of Industrial Engineering, University of Padova, 35131 Padova, Italy

^5^ Department of Laboratory Medicine, Medical University of Gdańsk, 80-210 Gdańsk, Poland

^6^ Department of Machine Design and Maintenance, AGH University of Kraków, 30-059 Kraków, Poland

^7^ Department of Manufacturing and Production Engineering, Gdańsk University of Technology, 80-233 Gdańsk, Poland

* Correspondence: balbina.makurat-kasprolewicz@pg.edu.pl; tel.: +48-58-347-14-65

Number of tables: 1
Number of descriptions: 1

Number of figures: 2

Number of references: 3

**Supporting Tables**

**Table S1.** The surface chemical composition (n=3) (by weight %) of coatings determined using EDS analysis.

| **Sample** | **Titanium** | **Zirconium** | **Niobium** | **Phosphorus** | **Calcium** | **Oxygen** |
| --- | --- | --- | --- | --- | --- | --- |
| 68_450_n_300 | 49.2±0.25 | 7.86±0.15 | 7.69±0.17 | 2.64±0.18 | 1.44±0.11 | 31.1±0.26 |
| 68_450_sin_300 | 50.6±0.58 | 7.98±0.13 | 7.93±0.20 | 2.19±0.30 | 1.42±0.18 | 29.7±0.99 |
| 136_450_n_300 | 49.21±0.17 | 7.75±0.14 | 7.67±0.12 | 2.64±0.11 | 1.42±0.03 | 31.3±0.29 |
| 136_450_rec_300 | 49.88±1.02 | 8.06±0.16 | 8.13±0.19 | 2.45±0.33 | 1.49±0.15 | 30.0±0.98 |
| 136_600_n_300 | 49.09±0.17 | 7.80±0.12 | 7.81±0.11 | 2.76±0.10 | 1.64±0.10 | 30.9±0.14 |
| 136_600_sin_300 | 49.80±0.80 | 8.02±0.09 | 8.11±0.19 | 2.47±0.28 | 1.60±0.12 | 30.0±0.68 |

All data are expressed as means ± SD. There is no statistically detected difference between samples in the groups in our study.

**Supporting Descriptions**

**Description S1:** Analysis and discussion of the results obtained in scratch studies

The scratch test was utilized to determine the adhesion of MAO and UMAO coatings to the Ti13Zr13Nb substrate, by analyzing penetration depth, friction force and coefficient, and acoustic emission, as well as inspecting scratch path micrographs at progressive force [1,2]. All tested specimens exhibited coating abrasion, but the substrate exposure point varied. The load results in initial cohesive cracks (Lc_1_) were observed in the 68_450_n_300 specimen (Figure S1a), while the highest critical load causes total damage of the coating (Lc_2_) was observed in the 136_450_n_300 specimen (Figure S1b). Comparison of the microstructures of the coatings after scratch tests showed no significant differences. Transverse cracks appeared at forces ranging from ~5.2 N to ~7.1 N, depending on the specimen. As the force increased, fragmentary peeling of the coating was observed in each sample. However, the literature suggests that isolated damages can be ignored [3]. Therefore, to accurately assess the upper critical load, which describes the adhesion strength of the coating to the substrate (Lc_2_ in Figure S1), microscopy analysis was used. The subsequent stages of coating destruction are well illustrated by the acoustic emission (AE) signal. Large changes in the AE signal were measured during coating cracking and abrasion. Above the critical load Lc_2_, when abrasion of the coating to the substrate occurred, AE is at a low level due to the cooperation of the indenter only with the metallic substrate.

**Supporting Figures**


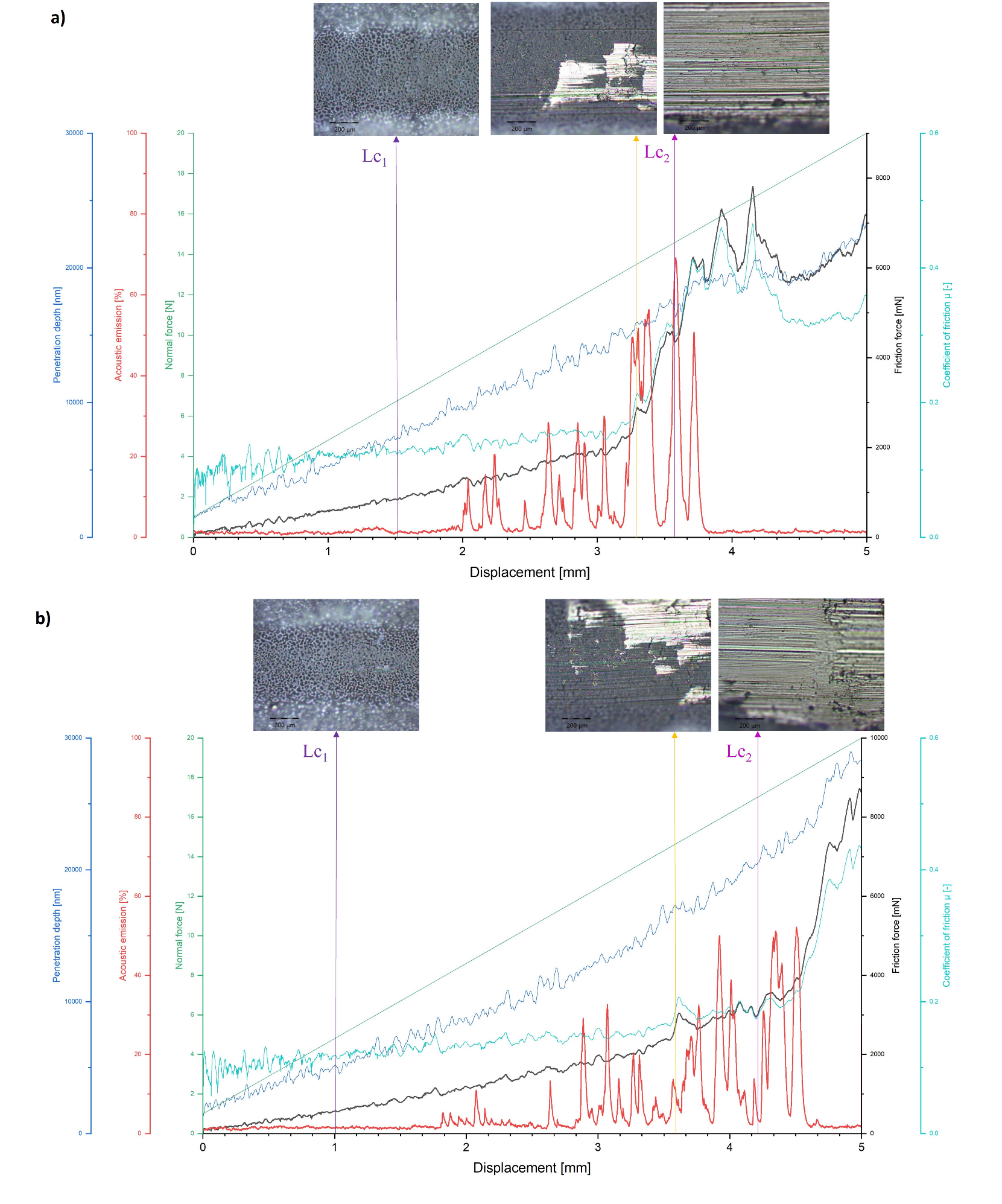


**Figure S1.** Images of the scratch path in places where characteristic coating damage occurs and the correlation between penetration depth, acoustic emission, friction force, and coefficient of friction with normal force, indicating the critical loads (Lc_1_ and Lc_2_), for the a) 68_450_n_300 and b) 136_450_n_300 specimens. The yellow arrow indicates the last partial abrasion. The results presented are representative of three tests.

**
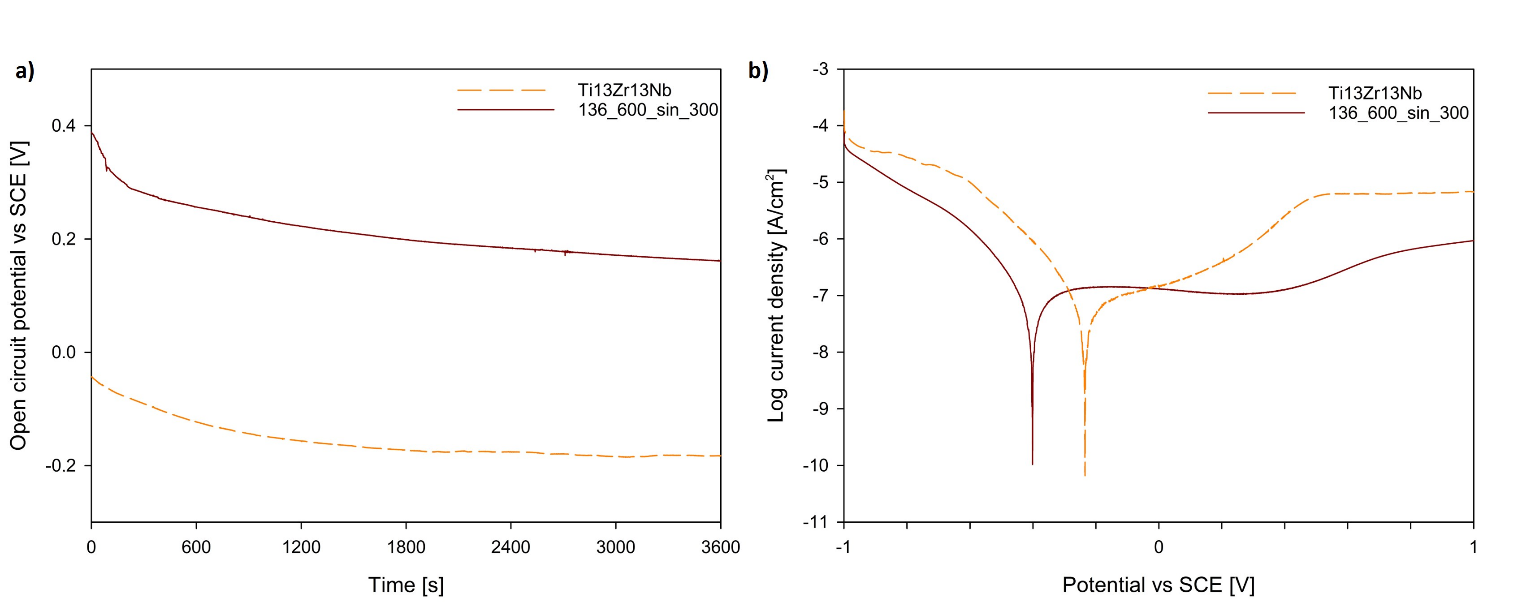
Figure S2.** a) Open-circuit potential vs. time and (b) potentiodynamic polarization curves recorded for uncoated Ti13Zr13Nb and 136_600_sin_300 specimens. The research was performed in Ringer’s solution at a scan rate of 0.1 mV/s at 37 °C. Surface modification slightly increased the corrosion current density of the biomaterial by ~17%. The presented results are representative of three analyses of each surface treatment specimen.

**References**

1. Yao, J.H. et al. (2019) Growth characteristics and properties of micro-arc oxidation coating on SLM-produced TC4 alloy for biomedical applications. Applied Surface Science 479, 727-737. 10.1016/j.apsusc.2019.02.142

2. Makurat-Kasprolewicz, B. et al. (2024) Influence of Ultrasound on the Characteristics of CaP Coatings Generated Via the Micro-arc Oxidation Process in Relation to Biomedical Engineering. ACS Biomaterials Science & Engineering. 10.1021/acsbiomaterials.3c01433

3. Randall, N.X. (2019) The current state-of-the-art in scratch testing of coated systems. Surface & Coatings Technology 380. 10.1016/j.surfcoat.2019.125092
