# Supplementary figures and images for: Effect of ultrasound on the physicochemical, mechanical and adhesive properties of micro-arc oxidized coatings on Ti13Nb13Zr bio-alloy
Source: Sci Rep. 2024 Oct 25;14:25421. doi: 10.1038/s41598-024-75626-4 (PMC11511822; doi:10.1038/s41598-024-75626-4)

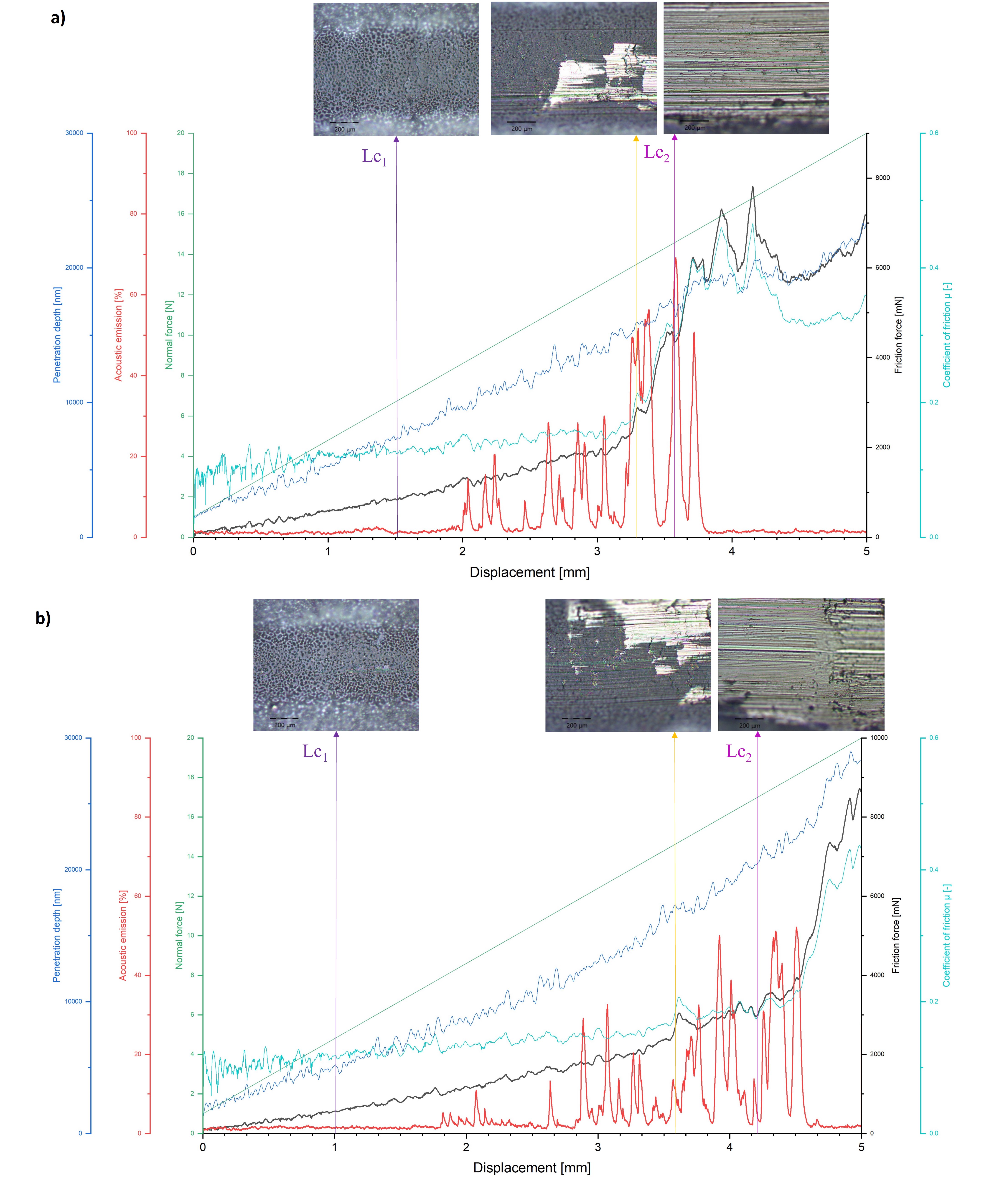

Supplement: Supplementary file 2 — Supplementary Material - Figure S1 [file 41598_2024_75626_MOESM2_ESM.jpg]

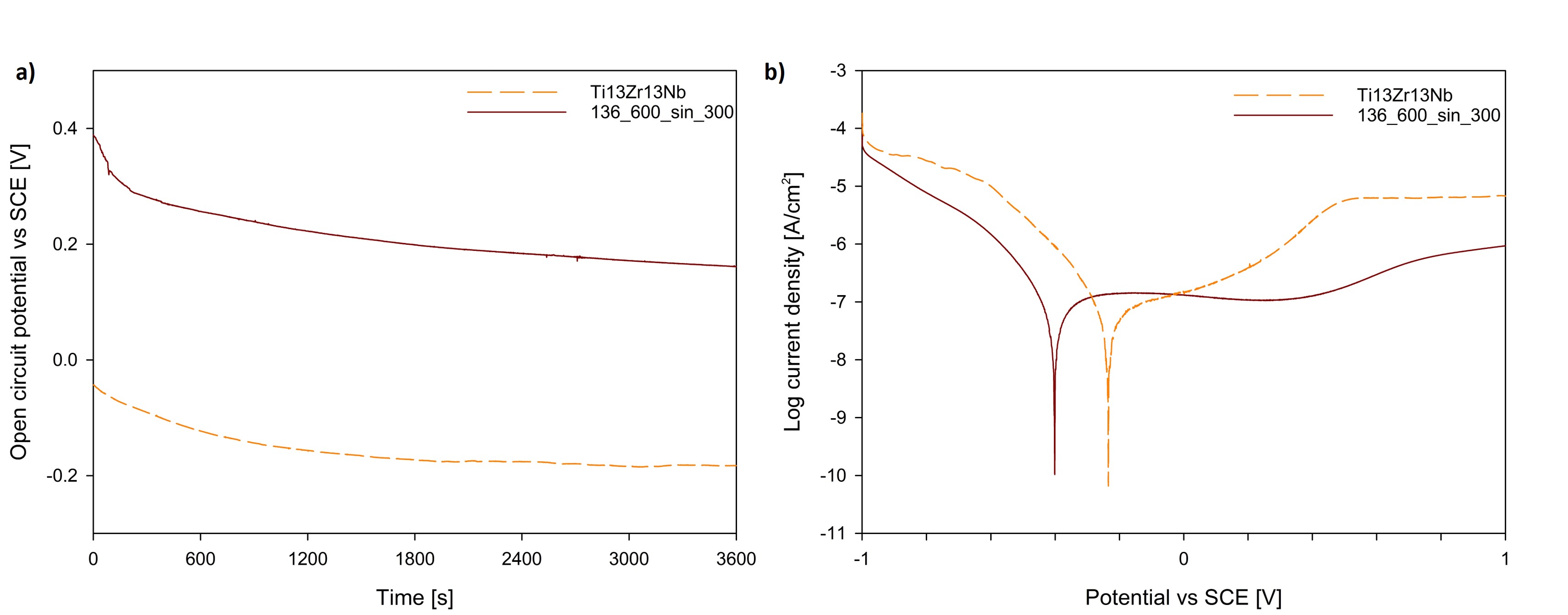

Supplement: Supplementary file 3 — Supplementary Material - Figure S2 [file 41598_2024_75626_MOESM3_ESM.jpg]
